# Prognostic and reproductive outcomes in women who had uterine myomas removed during cesarean section and sutured using different techniques

**DOI:** 10.1186/s12905-023-02852-9

**Published:** 2024-01-02

**Authors:** Qiao-Hong Dai, Lu Zhang, An-Er Chen

**Affiliations:** 1 Department of Gynecology and Obstetrics, Ningbo Women and Children’s Hospital, 339 Liuting Street, Haishu District, Ningbo, Zhejiang 315000 China; 2 Department of Internal Medicine, Ningbo Urology & Nephrology Hospital, Ningbo, 315000 China

**Keywords:** Bleeding, Cesarean section, Pregnancy with uterine myomas, Subsequent pregnancy, Suture in the pattern of a purse at the fundus, Tourniquet

## Abstract

**Background:**

In this study, the prognostic and reproductive outcomes of women who underwent excision of uterine myomas and were sutured using different techniques while undergoing a cesarean section were investigated.

**Methods:**

A total of 299 females who underwent cesarean section between January 2015 and June 2022 due to a scarred uterus were enrolled in this study. These participants were segregated into two categories: the experimental group (comprising 155 cases) in which uterine myoma (single lesion) was excised during the cesarean procedure, and the control group (consisting of 144 cases) in which only the cesarean section was conducted. A comparison between the two groups was carried out based on the following parameters: volume of intraoperative bleeding (mL), additional measures taken for intraoperative hemostasis (n, %), percentage (%) of patients experiencing postoperative fever, duration required for the passage of gas (hours [h]), length of hospital stay (days [d]), weight of newborns (kg) and their Apgar scores, and the reproductive outcomes of the experimental group assessed two years after the surgical procedure.

**Results:**

In the experimental group, the amount of bleeding during surgery, occurrence of postoperative fever among women, time taken for patients to resume passing gas, and length of hospital stay were 540.65 ± 269.12 mL, 9.03%, 15.99 ± 4.68 h, and 5.08 ± 1.18 days, respectively. In contrast, the control group had values of 409.03 ± 93.24 mL, 2.77%, 16.24 ± 4.92, and 4.47 ± 0.70 days, respectively (*P* < 0.05). No notable increase was observed in the need for additional intraoperative hemostasis measures, and there was no significant difference in the time it took for patients to pass gas after the surgery. All newborns had positive health status. In the experimental group, 25 patients underwent subsequent pregnancies, and 15 of them successfully reached full-term deliveries, all of which had positive outcomes.

**Conclusion:**

Combining myomectomy with various suture methods during cesarean delivery did not cause excessive bleeding and resulted in healthy newborns. This approach offers the advantage of avoiding additional surgeries under anesthesia and can be considered a viable option. Subsequent pregnancies after myomectomy were considered high-risk.

## Background

Uterine myomas are the most prevalent type of non-malignant growth of the female reproductive system. They exhibit a higher prevalence among expectant mothers and have emerged as one of the frequent complications associated with pregnancy. These growths are predominantly observed in pregnant women aged between 30 and 50 years, with approximately 20% of women above 30 years hosting uterine myomas. In this group, the incidence of uterine myomas during pregnancy varied from 0.3 to 3.0%. With advancements in accurate supplementary examinations and heightened health awareness among patients, the annual detection rate of uterine myomas during pregnancy has been progressively rising [1].

Females who have uterine myomas and do not intend to get pregnant again face the possibility of needing further surgery due to disruptions in their menstrual cycle, anemia, painful menstruation, and potential myoma growth-related complications. Those who wish to become pregnant are at a higher risk of experiencing symptoms caused by the pressure from an enlarged uterus, including issues like miscarriage, premature labor, excessive bleeding after childbirth, hormonal imbalances during pregnancy, and complications related to degenerative changes [2]. The practice of removing uterine myomas during a cesarean section has been a subject of debate for a while, as it is considered a challenging procedure that carries risks, including the potential for significant uncontrollable bleeding during surgery [3]. Presently, a clear and unanimous consensus regarding the possibility of excising myomas during cesarean section is lacking. Nevertheless, owing to the growing number of patients requiring this procedure, it has progressively become a routine practice among surgeons in the medical field and is regarded as a viable option. Emphasis is placed on comparative safety. In this study, 155 expectant mothers diagnosed with uterine myomas were enrolled, and their safety and reproductive results after myoma removal in conjunction with cesarean section were retrospectively analyzed.

## Methods

### Clinical data

Female patients who were admitted to the hospital between January 2015 and June 2021 for the purpose of undergoing a cesarean section to terminate their pregnancies, were enrolled in this study. All surgeries were performed by the same proficient obstetrician, who acted as the primary surgeon. The experimental group included 155 women who had a single myoma removed during cesarean section. These myomas were identified using ultrasound scans, with the smallest myoma diameter being > 4 cm and the largest being 16 cm. The control group consisted of 144 women who underwent a solitary cesarean section. In all cases, the women required cesarean section due to a previously scarred uterus, and the surgical plan was understood and agreed upon by both the women and their families. Participants in both the experimental and control groups were excluded if they met any of the following criteria: (1) Experienced bleeding before surgery: (2) Had conditions linked to blood disorders or increased bleeding susceptibility; (3) The patient was slated for additional gynecological procedures alongside uterine myoma removal during the cesarean Sect. 4. The patient is currently experiencing a fever attributed to COVID-19. This study was approved by the ethics committee, and the participants provided informed signed consent. Details of the basic information, surgery-related specifics, and post-surgery recovery status of the study participants were compiled by examining patient medical records and conducting telephone interviews.

### Surgery methods

All 299 female participants successfully completed the preparatory steps for the surgery. The procedure involved combined spinal-epidural anesthesia. Access to the abdominal area was gained through the original incision site. The specific steps are outlined as follows.

1. In the control group, conventional cesarean section was performed by making a transverse incision in the lower uterine segment. Following delivery, 20 units of oxytocin were administered to induce uterine contractions. Subsequently, the uterine incision was stitched, and the abdominal layers were closed.
2. In the experimental group, after the infants were delivered, 20 units of oxytocin were injected to stimulate uterine contractions. After removing the placenta, the uterus was positioned outside the abdominal incision to evaluate the location and type of the uterine myoma.

These myomas were categorized into eight different types, labeled as types 0 to 8 [4]. A tourniquet was then applied around the lower uterine segment, temporarily interrupting the uterine blood supply for one rotation.

The subsequent procedures were carried out according to the type of myoma, as detailed below.

**Fig. 1:**
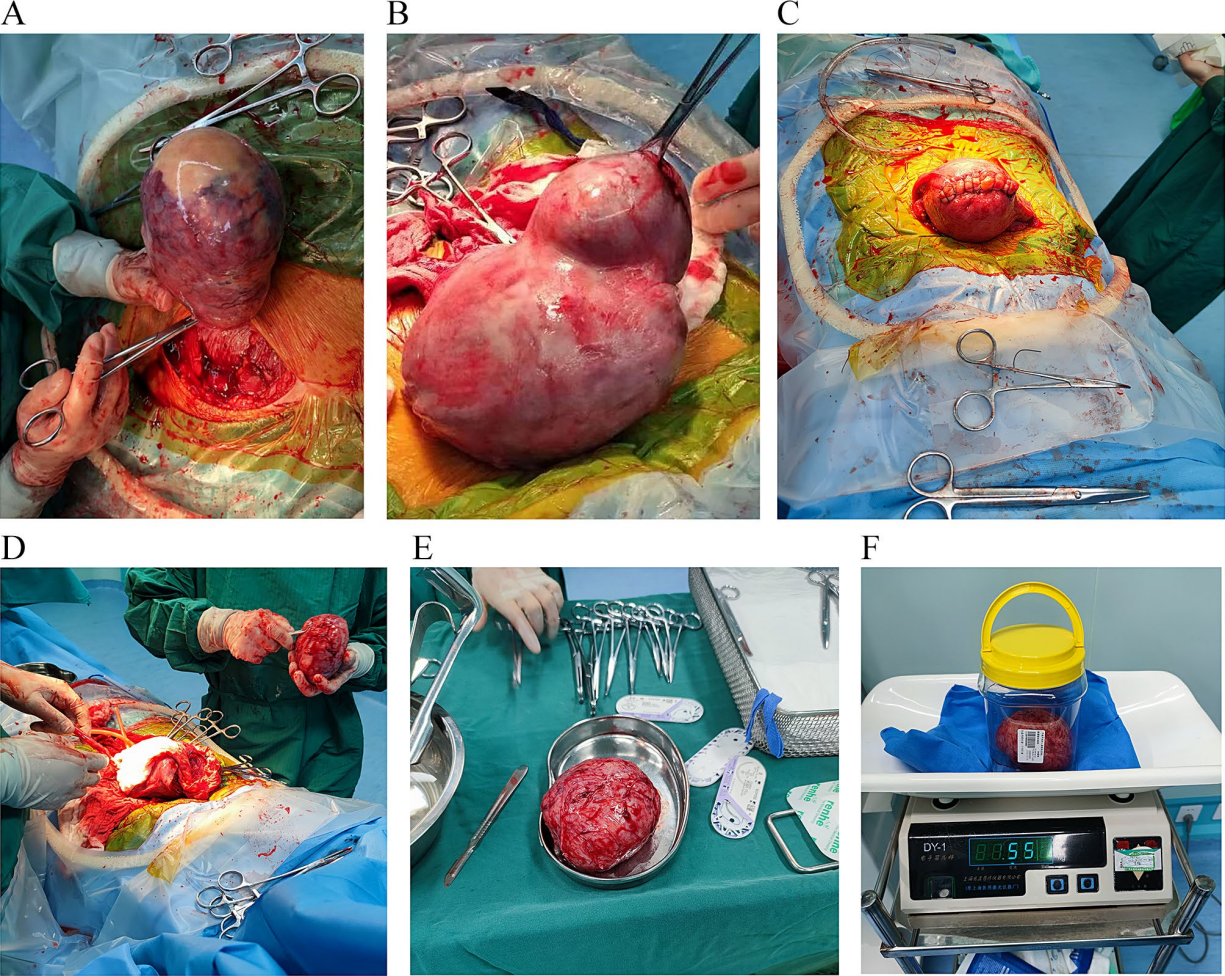
**A**: Myoma beneath serosa and with a pedicle; **B**: Preoperative myomas between muscular walls; **C**: Postoperative myomas between muscular walls; **D**: Excised myomas; **E**: Diameter of the myoma equal to 16 mm; **F**: The weight of myoma is 550 g

1. Myomas classified as Types 0 and 7 were encircled and tied off at their bases using threads before excision (Fig. 1A).
2. Myomas categorized as types 1–3 were accessed by passing through the uterine mucosa to reach the surface of the myoma. A straight incision was made in this region until the pseudocapsule was opened. The myoma was then grasped with a towel clamp and gently pulled outward. A circular purse-like stitching pattern using thread #0 was applied to the fundus to prevent empty space formation. The primary surgeon performed careful dissection of the myoma while the assistant tightened the stitching and secured a knot. The uterine cavity lining was stitched intermittently from the top of the incision using an absorbable thread (#1 − 0), and the mucosal layer was continuously sutured using an absorbable thread (#3 − 0) to restore the natural anatomy.
3. Uterine myomas falling under Types 4–6 were approached from the serosa layer adjacent to the myoma location. A linear incision was made in this manner (using a cold scalpel). The stitching technique was the same as described above (without penetrating the endometrium) to ensure the integrity of the uterine serosa (illustrated in Fig. 1B and C). The largest removed myoma was 16 cm in diameter (Fig. 1D, E and F).
4. For myomas categorized as type 8, the choice of incision method depended on their proximity to the mucosa or serosa layer. Near the mucosal layer, the second method described above was applied. Conversely, if close to the serosa layer (such as in cervical myomas), the third method described above was employed. Both groups received antibiotics of the same grade post-surgery to prevent infections, along with symptomatic treatment using uterine contraction agents. After surgery, the fibroid tissue was sent for routine pathological examination, which indicated uterine fibroids. During pregnancy, uterine fibroids are prone to degeneration (calcification, redness, hyaline transformation, cystic transformation, and sarcomatoid transformation). Twenty-three specimens were sent for rapid resection during surgery, which were all showed malignant transformation.

### Observation indicators

The two groups were compared based on intraoperative conditions: intraoperative bleeding volume (mL) and additional intraoperative hemostasis measures (n, %). Postoperative recovery conditions: proportion of women with postoperative fever (%), time spent passing gas (h), and hospitalization duration (d). Others: body weight of newborns (kg), Apgar scores, and reproductive outcomes of the experimental group two years after surgery.

### Statistical methods

The overall data and subsequent outcomes of all participants were statistically analyzed using SPSS 23.0. Statistical representation: Mean ± standard deviation was used for normally distributed measurement data, whereas rates were employed for enumeration data. Hypothesis testing: An independent samples *t*-test was used to compare the normally distributed measurement data between the two groups. For grouped enumeration data presented in fourfold tables, Pearson’s chi-squared test was used for comparison and assessment. A significance level of P < 0.05 indicated a statistically noteworthy distinction.

## Results

### General conditions

No statistically significant differences were observed between the two groups with respect to age, gestational age, gravidity, number of deliveries, and number of cesarean sections (*P* > 0.05) (Table 1).

**Table 1:** Comparison of general data between the two groups ($\bar{x}$±s)

Group	Age ($\bar{x}$±s, years)	Gestational weeks ($\bar{x}$±s, weeks)	Gravidity ($\bar{x}$±s, time)	Number of deliveries ($\bar{x}$±s, time)	Number of cesarean sections ($\bar{x}$±s, time)
Experimental group (n = 155)	34.35 ± 4.34	38.42 ± 0.85	2.86 ± 0.11	2.05 ± 0.22	2.05 ± 0.21
Control group (n = 144)	34.29 ± 3.74	38.42 ± 0.73	3.10 ± 1.08	2.06 ± 0.23	2.05 ± 0.22
t value	0.121	-0.046	-1.834	-0.151	-0.141
*P* value	0.091	0.475	0.855	0.763	0.779

### Intraoperative conditions

The intraoperative bleeding volume (in mL) exhibited a statistically significant difference between the two groups (*P* < 0.05), whereas there were no significant differences in other intraoperative hemostasis measures (such as case numbers and proportions) between the groups (*P* > 0.05) (Table 2). The mean time of uterine fibroids resection was 8.34 ± 4.38 min (range: 3–23 min).

**Table 2:** Comparison of intraoperative conditions between the two groups

Group	Bleeding volume ($\bar{x}$±s, mL)	Additional intraoperative hemostasis measures (n, %)			Hysterectomy (n, %)
		Intrauterine balloon tamponade	Ligation of uterine artery ascending branch	Uterine artery embolization	
Experimental group (n = 155)	540.65 ± 269.12	5(3.23)	4(2.58)	0(0.00)	0(0.00)
Control group (n = 144)	409.03 ± 93.24	4(2.78)	3(2.08)	0(0.00)	0(0.00)
t value	5.566	-	-	-	-
*P* value	0.000	0.821	0.776	-	-

### Postoperative recovery conditions

The time taken for gas passage was not significantly different between the two groups (hours) (*P* > 0.05). However, significant differences were observed in terms of hospitalization duration (days) and proportion of women experiencing fever (*P* < 0.05). None of the groups recorded instances of reoperation or mortality (Table 3).

**Table 3:** Comparison of postoperative recovery conditions between the two groups

Group	Time for passing gas ($\bar{x}$±s, h)	Hospitalization duration ($\bar{x}$±s, d)	Fever (n, %)	Another surgery (n, %)	Death (n, %)
Experimental group (n = 155)	15.99 ± 4.68	5.08 ± 1.18	9.03	0	0
Control group (n = 144)	16.24 ± 4.92	4.47 ± 0.70	2.77	0	0
t value	-0.449	5.406	-	-	-
*P* value	0.611	0.001	0.023	-	-

### Conditions of newborns

Infants from both categories exhibited no statistically significant differences in newborn weight or Apgar scores (*P* > 0.05) (Table 4).

**Table 4:** Comparison of outcomes for newborn between the two groups

Group	Body weight of newborns ($\bar{x}$±s, kg)	Apgar scores (1 min)	Apgar scores (5 min)	Death of newborns
Experimental group (n = 155)	3.32 ± 0.40	8.97 ± 0.16	10.00 ± 0.00	0
Control group (n = 144)	3.34 ± 0.38	8.99 ± 0.12	10.00 ± 0.00	0
t value	-0.445	-0.732	-	-
*P* value	0.199	0.142	-	-

## Discussion

Owing to the relaxation of birth policies and advancements in medical care, the identification rate of uterine myomas during pregnancy has progressively risen. As individuals seek better postpartum quality of life and physical health, addressing the management of uterine myomas during childbirth has become a pressing concern. Some experts, such as Ruan et al. [5], argue against prioritizing the treatment of uterine myomas during pregnancy unless conservative approaches are ineffective under unfavorable circumstances, such as myoma pedicle torsion, myoma incarceration, uterine torsion, or myoma red degeneration. Their viewpoint stems from the understanding that hormonal influences on the uterus during pregnancy can lead to enlarged myomas with indistinct boundaries. Furthermore, the uterine tissue is more relaxed and vascular, complicating surgery and elevating risks like postpartum bleeding, infection, and even intensive care unit admissions [6]. They propose a secondary surgery to remove myomas once the uterus has reverted to its normal state [7].

In contrast, some scholars contend that the pregnant uterus responds well to oxytocin, and bleeding during cesarean section is typically manageable. Performing myomectomy concurrently with a cesarean section could assuage patient concerns about future wellbeing and sidestep the pain and financial burden of a separate surgery, as demonstrated in 405 cases by Shang et al. [8] As a seasoned clinical practitioner with more than a decade of experience, I confront this issue directly. My aim is to curtail the extra expenses of subsequent myoma removal procedures, prevent repetitive exposure to anesthesia, and shield patients from complications linked to uterine myomas, such as secondary postpartum hemorrhage, subpar uterine recovery, anemia, infection, myoma-related pressure, and even malignant transformation [9]. The study also outlined recuperation conditions post-surgery, including outcomes in subsequent pregnancies.

The two groups of participants in this study did not display significant differences in terms of age, number of pregnancies, number of deliveries, timing of pregnancy termination, instances of cesarean sections, newborn weights, and Apgar scores. Myomectomy surgeries for uterine fibroids in this study group were all performed after the newborn delivery, so it had no impact on the postpartum condition of the newborn. the mean time spent on resection of myomas was 8.34 ± 4.38 min (range 3–23 min). Proficient surgical procedures and techniques reduce the overall surgical time not very long. Although there was an increase in bleeding volume in the experimental group, this increase did not result in any significant increase in the proportion of women requiring further hemostasis measures during surgery, nor did it lead to uncontrollable excessive bleeding, heightened risks of subsequent surgeries, or mortality. While there were variations in the rate of postoperative fever and length of hospital stay after surgery, these differences between the two groups were not statistically significant.

Postoperatively, there were no occurrences of intestinal obstruction or paralysis in either group, and there was no significant difference in the time taken for the passage of gas between the two groups. These outcomes can be attributed to the primary surgeon’s meticulous hemostasis techniques and careful efforts to restore the anatomical structure, particularly the continuity of the serosa and mucosa layers, while minimizing dead space [10, 11].

In this study, several noteworthy highlights emerged from the experimental group that deserve attention.

One notable highlight of this study is the use of a tourniquet to temporarily obstruct the lower segment of the uterus, thus effectively restricting the blood supply to the uterine artery. This minimally invasive method demonstrated visible effectiveness in achieving hemostasis. Additionally, in the control group, alternative hemostasis methods were employed, with nine women utilizing other remedial measures. No instances of uncontrollable postoperative bleeding or hysterectomy have been reported.

Another significant highlight of this study is the meticulous dissection of myomas and careful suturing of the wound surface, particularly through access to the endometrium. This approach aims to preserve the integrity of the serosa layer without altering the structure of the uterus, resulting in reduced pelvic adhesions. These practices were consistent with the findings reported by Huang et al. [12]. While most instances of wound surface bleeding were addressed through intrauterine balloon tamponade combined with other hemostasis methods, 23 women in the experimental group underwent myoma removal from the mucosal layer incisions. Among them, five received intrauterine balloon tamponade in addition to other hemostasis methods. Importantly, none of these patients experienced secondary postpartum hemorrhage, uterine cavity infections, or adverse outcomes.

Remarkably, thorough monitoring and evaluation were conducted for women aiming for subsequent pregnancy. Muscle layer thickness was assessed using magnetic resonance imaging (MRI) at specific intervals: 42 days, 3 months, 6 months, 1 year, and 2 years post-surgery. Among experimental group, 25 subsequent pregnancies were recorded. Of these, 15 cases opted for a full-term cesarean section, while four cases terminated pregnancies at 34 to 36 weeks due to obstetric complications. Three pregnancies were terminated at 32–34 weeks due to complications, and three ended in miscarriage. Notably, uterine rupture, severe postpartum bleeding, embedded placenta, or uterine removal did not occur. However, one case exhibited significant pelvic adhesion during cesarean section due to exudation and inflammation from a previous myomectomy procedure. In cases of excessively thin local muscle layers found in auxiliary examinations, proactive termination of pregnancy is imperative. In the control group, there were 36 cases of re-pregnancy, 24 cases of termination of pregnancy by full-term cesarean section, 8 cases of termination of pregnancy at 34–36 weeks (3 cases due to fetal hypoxia and 5 cases with obstetric complications such as hypertension), 2 cases of termination of pregnancy at 32–34 weeks (with obstetric complications), and 2 cases of miscarriage. During the operation, one patient was found to have placental implantation, and no patient had uterine rupture, severe postpartum hemorrhage, or hysterectomy. According to the two current sets of data, only retrospective observations can be made. The control group had a slightly higher rate of full-term childbirth than the study group, and one patient in the study group had severe pelvic adhesions. This is precisely because adhesions occurred at the site where uterine fibroids were removed during the previous cesarean section, but the sample size was small, which is not sufficient to prove the differences between the two groups. Therefore, it is necessary to expand the sample size. Currently, based on retrospective observations, the small data indicate that the two groups have good outcomes in reproducing after surgery, and there are no cases of endangering the life of the mother and fetus due to uterine rupture.

It is also worth noting the probability of uterine sarcoma in these patients, although its incidence is extremely low [13], and there was no uterine sarcoma in this study. The diagnosis and treatment of patients with misdiagnosis or missed diagnosis of uterine sarcoma during surgery, as well as the advantages and disadvantages of this surgery, should be explored in the future.

This study has several limitations. First, this was a retrospective analysis with an unavoidable bias. Second, this was an observational study that involved a single cohort. Thus, all results in the present analysis should be interpreted cautiously, randomized controlled trials with large sample sizes should be conducted, and the surgical approach and reproduction of these patients should be explored further.

## Conclusions

In conclusion, performing myomectomy alongside cesarean section offers a valuable opportunity to avoid repeated anesthesia exposure and additional surgical resources. The use of a tourniquet and meticulous decision making in selecting appropriate excision and suturing methods contribute to reduced pelvic adhesions. Supplementary measures effectively controlled the bleeding during the procedure. The overall prognosis is favorable, and it alleviates postpartum pressure. Subsequent pregnancies were treated with caution, resembling high-risk pregnancy management. Vigilant monitoring and increased prenatal examinations aimed to mitigate myoma-related complications during pregnancy and attain successful birth outcomes. However, comprehensive data on subsequent pregnancies after surgery are limited, emphasizing the importance of further large-scale studies and continued monitoring for comprehensive analysis.

## Data Availability

The data that support the findings of this study are available from the corresponding author, upon reasonable request.
